# A catastrophic seronegative anti-phospholipid syndrome: case and literature review

**DOI:** 10.1186/s12959-021-00356-w

**Published:** 2021-12-20

**Authors:** Vanda Pinto, Augusto Ministro, Nuno Reis Carreira, Ana Cardoso, Catarina Sousa Gonçalves, Mickael Henriques, João Rato, Emanuel Silva, Luís Mendes Pedro

**Affiliations:** 1 Vascular Surgery Department, Heart and Vessels Division, Hospital Santa Maria (CHULN), Av. Prof. Egas Moniz s/n, 1649-035 Lisbon, Portugal; 2 Lisbon Academic Medical Center, Lisbon, Portugal; 3 Faculty of Medicine, University of Lisbon, Lisbon, Portugal; 4 Department of Internal Medicine (Medicina 2), Hospital Santa Maria (CHULN), Lisbon, Portugal

**Keywords:** Antiphospholipid syndrome, Aortic thrombosis, Renal thrombosis

## Abstract

**Background:**

Antiphospholipid Syndrome (APS) is a multisystemic autoimmune disease characterized by arterial and venous thrombosis and / or obstetric morbidity in the presence of at least one circulating anti-phospholipid antibody. The spectrum of vascular events varies from deep venous thrombosis to catastrophic APS, a rare form characterized by acute multiorgan thrombosis and high mortality.

**Case report:**

We present the case of a 32-week pregnant woman arriving in the hospital emergency room with bilateral acute lower limb ischemia. In the obstetric evaluation, fetal death was declared. Computerized Tomography angiography showed pulmonary embolism of both pulmonary arteries, areas of splenic and right renal infarction and multiple arterial and venous thrombosis. The patient underwent urgent caesarean section and axillary-bifemoral bypass. No events registered. In the postoperative period, in an intensive care unit, treatment with rituximab and plasmapheresis were added to anticoagulant therapy. The laboratorial investigation was negative for thrombophilia and autoimmune diseases.

**Conclusion:**

Catastrophic APS develops quickly, with multiorgan involvement and high mortality rate. The presented case poses a multidisciplinary challenge, with the surgical approach of extra-anatomical revascularization being less invasive and guaranteeing immediate perfusion of the lower limbs. Although the serological tests were negative for anti-phospholipid antibodies, this case hardly fits into another diagnosis. Therefore, it was treated as a catastrophic APS, having shown a favorable evolution.

## Introduction

Pregnancy is characterized by a hypercoagulable state which confers protection against hemorrhagic challenges such as childbirth or miscarriage. Nonetheless, it simultaneously increases 4- to 5-fold the risk of thrombotic events [1], with the majority (75–80%) of pregnancy-related thrombotic events being venous. The most important risk factors are a previous history of thrombosis and thrombophilia, the most significant of which is antiphospholipid syndrome (APS) [1].

APS is a systemic autoimmune disorder (AID) characterized by recurrent arterial or venous thrombosis and/or pregnancy morbidity (fetal loss and stillbirth) in the presence of anti-phospholipid (aPL) antibodies, typically lupus anticoagulant (LA), anticardiolipin (aCL) or anti-β2 glycoprotein-I (β2GPI) [2–6]. APS can be secondary (associated with other diseases, mainly systemic lupus erythematosus) or can be found in patients with neither clinical or laboratory evidence of another condition (primary APS) [2, 5]. The APS presentation spectrum varies in severity and ranges from asymptomatic “carrier” for aPL to a life-threatening form, catastrophic APS (CAPS) [5, 7].

Catastrophic APS, also known as Asherson’s syndrome, is a rare condition (less than 1% of APS patients) which includes multiple organ system thromboses and develops over a short period with high mortality [6]. CAPS classification criteria (Table 1) requires thrombotic events in three or more organs, developing in less than a week; microthrombosis evidence in at least one organ; and persistent aPL positivity [5, 6]. Most cases of CAPS present as a microangiopathic storm rather than large-vessel thrombosis, however any combination of vascular occlusive events may occur in the same patient leading to multiorgan failure with diverse primary clinical manifestations, depending on the organ systems involved [5, 6, 8].

**Table 1:** Criteria for the classification of catastrophic APS^5^

1. Evidence of involvement of three or more organs, systems and/or tissues^a^	
2. Development of manifestations simultaneously or in less than a week.	
3. Confirmation by histopathology of small vessel occlusion in at least one organ or tissue^b^	
4. Laboratory confirmation of the presence of antiphospholipid antibodies (lupus anticoagulant and/or anticardiolipin antibodies)^c^	
*Definite catastrophic APS:* All 4 criteria.	
*Probable catastrophic APS:*	
– All 4 criteria, except for only two organs, systems and/or tissues involvement.	
– All 4 criteria, except for the absence of laboratory confirmation at least 6 weeks apart due to the early death of a patient never tested for aPL before the catastrophic APS.	
– Criteria 1, 2 and 4.	
– Criteria 1, 3 and 4 and the development of a third event in more than a week but less than a month, despite anticoagulation.	

^a^ Usually, clinical evidence of vessel occlusions, confirmed by imaging techniques when appropriate. Renal involvement is defined by a 50% rise in serum creatinine, severe systemic hypertension (> 180/100 mmHg) and/or proteinuria (> 500 mg/24 h)

^b^ For histopathological confirmation, significant evidence of thrombosis must be present, although vasculitis may coexist occasionally

^c^ If the patient had not been previously diagnosed as having an APS, the laboratory confirmation requires that presence of antiphospholipid antibodies must be detected on two or more occasions at least 6 weeks apart (not necessarily at the time of the event), according to the proposed preliminary criteria for the classification of definite APS

Because CAPS is so uncommon, an international registry was formed by the European Forum on Anti-Phospholipid Anti-bodies in 2000 and most recent reported data (2016) includes 500 patients [7].

Mortality rate has decreased over time mainly due to triple therapy (anticoagulation, corticotherapy and therapeutic plasma exchange - TPE - or intravenous immune globulin IVIG), but it remains still above 30% [7].

Our objective is to report an extremely rare and potentially lethal condition.

## Case report

A 38-year-old woman, 32^+ 2^ weeks pregnant, previous smoker with a past history of one miscarriage and livedo reticularis was admitted to the emergency department (ED) of her local hospital due to a sudden onset of pain, cold and functional impotence of the lower limbs. During the obstetric evaluation, fetal death was observed.

**Fig. 1:**
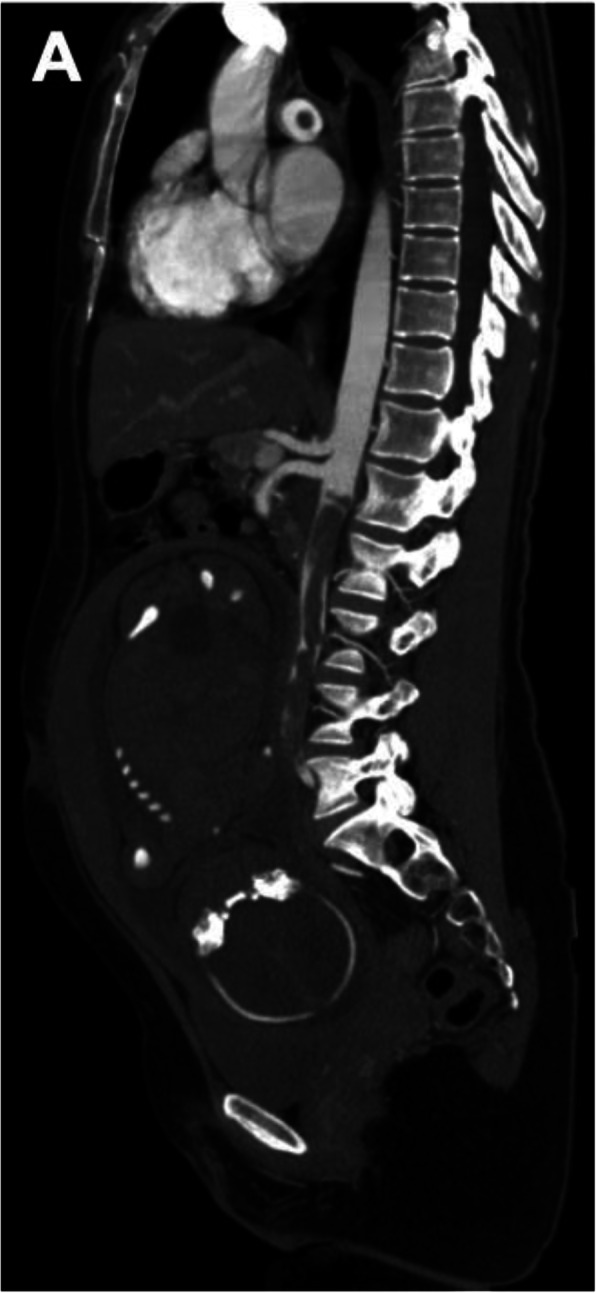
Angio-CT - sagittal view - showing juxta renal aortic thrombosis

**Fig. 2:**
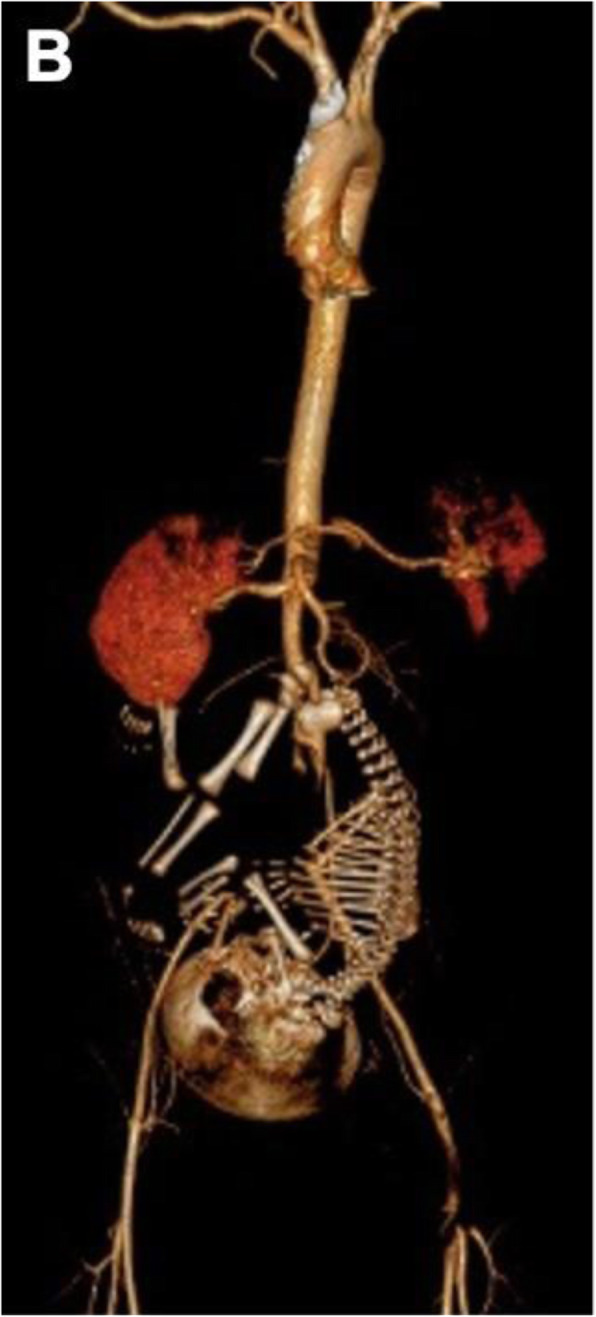
3D reconstruction of CT angiography showing areas of splenic and right renal infarction, thrombosis of the juxta renal aorta and left renal artery, the distal segment of the right deep femoral artery and the left common femoral artery and its bifurcation

Given the suspicion of lower limb ischemia, low weight molecular heparin was started, and the patient was transferred to our hospital, which has vascular surgery. On admission in our ED, she was alert and oriented, hemodynamically stable and presented with tachypnea. Absence of pulses, cold and pallor of the lower limbs, with minimal neurosensory deficit and muscle weakness were observed. Laboratory workup showed hypocapnia, thrombocytopenia, elevation of liver and pancreatic enzymes, elevated total creatinine kinase (CK) and lactate dehydrogenase (LDH). Table 2 resumes clinical and laboratory evolution during hospitalization. The Computed Tomography angiography (Angio CT) showed bilateral (central and lobar) pulmonary embolism (PE), deep venous thrombosis of the inferior vena cava and left iliac axis, areas of splenic and right kidney infarction and multiple arterial and venous thrombosis. Juxta-renal aortic thrombosis (Fig. 1) was also observed as well as thrombosis of the left renal artery (with hypocaptation of the left kidney), right common iliac artery, left hypogastric artery, left common femoral artery, right deep femoral artery and right tibioperoneal trunk (Fig. 2).

**Table 2:** Evolution of CAPS in the described patient

Organ manifestation	Clinical signs	Laboratory abnormalities	(reference range)	Laboratory tests which were normal (negative)
Kidney Renal Failure Renal infarction Renal artery thrombosis Lung Bilateral Pulmonary Embolism Liver Elevated liver enzymes Peripheral vessel Venous thrombosis Arterial thrombosis Spleen Splenic infarction Heart Obstetric fetal death	Polypnea Absence of pulses in the lower limbs Minimal neurosensory deficit Moderate muscle weakness	Hemoglobin: 11,1 g/dL WBC: 12,20 × 10^9/L Platelet count 78 × 10^9/L pH 7.37 pCO_2._ 20,5 mmHg HCO_3_^−^ 18,5 mmol/L Serum creatinine 1,09 mg/dL AST 106 U/L ALT 34 U/L Pancreatic amylase 98 U/L LDH 950 U/L Total Creatinine Kinase (CK) 1217 U/L D-dimers (DD) 4,72 μg/ml T-Troponin 2137 ng/L NT-proBNP 4041 pg/mL Proteinuria 75 mg / dL Leukocyturia 100 cel / uL Erythrocyturia 150 cel / uL	12.0–15.3 4.0–11.0 150–450 × 10^9/L 7,35 - 7,45 35–45 mmHg 22–26 mmol/L 0,5–9,0 mg/dL 0–32 U/L 0–33 U/L 13–53 U/L 100–250 U/L 26–192 U/L 0–0,5 μg/ml < 14 ng/L < 300 pg/mL NEGATIVE NEGATIVE NEGATIVE	Peripheral blood smear (showed no schizocytes) INR: 0,97 aPTT: 32,4/29 Fibrinogen: 397 mg/dL (200–400) Lupic anticoagulant aCL IgG; aCL IgM aβ2GPI IgG; aβ2GPI IgM Antinuclear antibodies Anti-DS-DNA antibodies Anti-cytoplasm (MPO; PR3) antibodies Anti-mitochondria antibody (AMA) Anti-smooth muscle antibody (ASMA) anti-citrulline antibody (anti-CCP) Rheumatoid factor HLA - B27 normal Complement count (C3; C4) Absence of Leiden Factor V mutation Absence of prothrombine mutation PT20210A Protein C and protein S Funcional Antithrombin III Anti-HIV 1/2 (CHIV Ag/Ab) Ag-HBs Anti-HCV SARS-CoV-2

As lower limb ischemia was tolerated (acute limb ischemia - grade IIA), surgery was postponed. The patient was admitted to the intensive care unit (ICU) where presumptive CAPS diagnosis was made and anticoagulation with unfractioned heparin infusion was started. Despite anticoagulation, the patient presented worsening of neurosensory deficit and muscle weakness (acute limb ischemia - grade IIB), with the need of urgent revascularization surgery. Under general anesthesia, a cesarean section was performed to extract the dead fetus, and intrauterine balloon tamponade was placed for control of postpartum hemorrhage, followed by bilateral thromboembolectomy of the lower limbs and axillary-bifemoral bypass with synthetic prosthesis. Surgery was uneventful. Fetus had no anatomical abnormalities and weighed 1540 g. The patient recovered both lower limb perfusion as well as bilateral femoral, popliteal and distal pulses and was extubated the next day. During her stay in the ICU, the patient maintained therapeutic anticoagulation, underwent a total of six sessions of plasmapheresis and the first dose of rituximab (1000 mg) was administered. Under this treatment, an evident improvement was observed: the platelet count, hepatic enzymes and serum creatinine normalized. On the sixth day of hospitalization, she was transferred to an intermediate care unit at the hospital of her residence area where she continued TPE until the 14th day, when the second dose of rituximab was administered. After those treatments, the patient was discharged with a vitamin K antagonist. Regarding the initial laboratory workup, before plasmapheresis or rituximab infusions, antiphospholipid antibodies were negative (LA, aCL and β2GPI) as well as screening for thrombophilia (including protein C and protein S deficiency, antithrombin III deficiency, Leiden Factor V mutation and prothrombin mutations), and other autoimmune diseases. Histological examination of the placenta showed areas of small vessel infarction.

Six months after discharge, the patient has resumed her day-to-day life activities. She remains under warfarin therapy and the bypass is functional, with distal pulses maintained and without intermittent claudication. Additional workup was repeated and continued to be negative for aPL (even with the patient taking warfarin, which could cause a false positive lupus anticoagulant) and other autoimmune diseases (including SLE, vasculitis and sarcoidosis), with normal complement counts (C3, C4 and CH50) as well as negative for thrombophilia, cryoglobulinemia, cold agglutinin disease, autoimmune hemolytic anemia, paroxysmal nocturnal hemoglobinuria and infectious diseases (negative for hepatitis B virus, hepatitis C virus, cytomegalovirus, Epstein Barr and toxoplasmosis). CD19 positive cells are still depleted (0,1%; normal 7–23%), due to previous rituximab infusions. Other extra-criteria antibodies testing was not available in the laboratory and was not performed [Table 3].

**Table 3:** Suggested extra-criteria antibodies in seronegative antiphospholipid syndrome and its clinical manifestations. (Adapted from [9])

Extra-criteria antibodies	Clinical manifestations
Anti-prothrombin/phosphatidylserine antibodies	Thrombosis
Anti-annexin V antibodies/annexin A5 resistance	Thrombosis and/or pregnancy complications
Antibodies to vimentin/CL complex	Arterial thrombosis
Phosphatidylethanolamine	Fetal loss and/or thrombosis
Phosphatidic acid	Fetal loss
Phosphatidylserine	Fetal loss
Phosphatidylinositol	Fetal loss

## Discussion

The initial diagnosis of CAPS was clinical, given the sudden onset of thrombosis in large and small vessels, and across multiple organs, as clearly shown on Angio CT. Her previous miscarriage, livedo reticularis and thrombocytopenia might support a previous undiagnosed APS and the initial trigger might have been the pregnancy, that culminated in fetal death, which is the precipitating factor in 8% of CAPS registry cases [7].

Differential diagnoses included other entities, such as HELLP syndrome, disseminated intravascular coagulation (DIC) and malignancy. HELLP syndrome was not suspected since hyperbilirubinemia was not present and peripheral blood smear did not show signs of microangiopathy, including the absence of schizocytes. There was no evidence of DIC because coagulation times were not prolonged, fibrinogen was not reduced (397 mg/dL [200–400]). There was no suspicion of malignancy since she had no signs or symptoms of neoplastic disease, and no suspected features were found in the CT scan.

As a condition that is both rare and potentially lethal, there are no randomized clinical trials to guide CAPS treatment with the recommendations being empiric: based on analysis of CAPS registry and expert consensus [6, 10]. “Triple therapy” is the most accepted treatment at date and consists of anticoagulation, corticotherapy and therapeutic plasma exchange (TPE/plasmapheresis) or intravenous immune globulin (IVIG). New therapeutic modalities such as rituximab may have a role as a second-line therapy in the treatment of complicated, refractory/relapsing CAPS patients [6, 10]. Additionally, potential initiating factors or “triggers” must be identified and treated, and supportive care must be given as for any critically ill patient [6, 10, 11].

In the present case report, thrombolysis wasn’t indicated as treatment for the PE, since the patient was hemodynamically stable and would probably need surgery in a few hours. Anticoagulation with unfractioned heparin infusion was started in the ICU, to allow rapid reversal if surgery was required and, for its additional anti-inflammatory effects (especially inhibition of complement activation and inhibition of aPL binding) [6, 10, 12]. Despite all efforts, lower limb ischemia continued to worsen, and revascularization became urgent. Several surgical options were considered. Endovascular surgery was excluded due to the high risk of in-stent thrombosis. Anatomical revascularization surgery, despite presenting better long-term permeability, presented a high hemorrhagic risk and the presence of a gravid uterus was an anatomical concern. Therefore, we opted for a less invasive extra-anatomical revascularization (axillary-bifemoral bypass) to ensure immediate perfusion of the lower limbs, postponing an eventual anatomical revascularization for a second time (after stabilization). Considering the risk of anastomotic blow out and bypass thrombosis with an induction of labor (due to effort and anatomical position of the delivery), a cesarean section was performed. An intrauterine balloon tamponade was placed to minimize the bleeding risk after cesarean section and was removed after 48 h.

Immunosuppression with corticosteroids inhibits the cytokine cascade, which plays a major role in CAPS [6, 11], but it also delays the healing process and increases the risk of surgical wound and bypass prosthesis infection. For this reason, rituximab, a chimeric monoclonal antibody against CD20 that reduces the number of B- cells and therefore the production and concentration of harmful cytokines, was initiated instead. TPE was used for additional immunosuppression since it removes circulating free antibodies (aPL), immune complexes, cytokines, TNF- α, and complement products [11, 13].

During hospitalization, the laboratorial results for aPL were negative, and it was assumed that were false negatives due to consumption [8] or the presence of other aPL antibodies not usually tested [14].

During the six-month follow-up after discharge, laboratory workup persisted negative for autoimmune diseases, thrombophilia or infectious disease. However, CD19 positive cells remained depleted (0,1%; normal 7–23%) due to rituximab infusions, which could explain a negative aPL, with or without other autoimmune disease. Another possibility is the existence of other aPL besides LA, aCL and β2GPI, that aren’t routinely measured in most laboratories.

Given the rapid development of manifestations, the number of organs involved and the histopathological confirmation of small vessel occlusion in the placenta, this case undoubtedly fulfills criteria 1, 2 and 3 for CAPS diagnosis (Table 1). However, the absence of positive aPL prevents this diagnosis, even though this case hardly fits any other known coagulopathy, or diagnosis.

In clinical practice, there are often discrepancies between antibody levels and clinical disease expression. Routine screening tests for aPL may miss some cases and it is also possible that previously positive aPL titers become negative - either acutely by “consumption” during an acute thrombotic episode, or slowly, over time [8, 15]. In 2003, Hughes and Khamashta introduced seronegative APS (SNAPS) to describe patients with clinical manifestations highly suggestive of APS but with persistently negative aPL [15]. Since then, other reviews recognizing this entity were published [8, 9, 14, 16, 17] and some new antigenic targets for aPL in APS have been recently investigated, some of them associated with thrombosis and/or fetal loss [9] (Table 3 [9]).

SNAPS is usually a diagnosis of exclusion and should be suspected in patients with a clinical history suggestive of APS, such as those with recurrent arterial venous thrombotic events, recurrent miscarriage, or unexplained thrombocytopenia, with persistent negativity of aPL tested on at least two occasions, and when other causes of thrombosis are excluded, such as thrombophilia, active cancer, trauma, major surgery, or prolonged bed rest [9]. SNAPS diagnosis implies fulfilling clinical criteria, plus “non-criteria” manifestations, with persistently negative aPL [18]. “Non-criteria” manifestations are described in Table 4 [9, 18]. In this case, it stands out the presence of livedo reticularis and thrombocytopenia as a “non-criteria” manifestation of antiphospholipid syndrome.

**Table 4:** “Non-criteria” manifestations of antiphospholipid syndrome (adapted from [9, 18])

Nervous system	Dementia
	Seizures
	Multiple sclerosis–like illness
	Chorea
	Myelitis
	Brain MRI white matter lesions
	Cognitive impairment
	Migraine
Skin	Livedo reticularis
	Livedoid vasculopathy
	Raynaud’s phenomenon
	Splinter hemorrhages
	Skin ulcers
Heart	Valve vegetations or thickening (Libman-Sacks Endocarditis) Diastolic dysfunction
Blood	Thrombocytopenia
	Hemolytic anemia
	Coombs´ test positivity
Kidney	Microangiopathy
	Chronic vaso-occlusive lesions (atherosclerosis, glomerular ischemia, interstitial fibrosis, arterial fibrous intimal hyperplasia)
Obstetric	Late intrauterine growth restriction (after 34 weeks)
	Late pre-eclampsia (after 34 weeks)
	Placental abruption
	Placental hematoma
	Preterm birth (> 34 to < 37 weeks)
	Puerperal pre-eclampsia
	Two or more unexplained in vitro fertilization failures
	Two unexplained spontaneous abortions < 10 weeks
Other	Superficial vein thrombosis
	Amaurosis fugax
	Sensorineural hearing loss
	Ischemic necrosis of bone
	Pulmonary hypertension

This patient could be either a truly SNAPS (in the absence of extra-criteria antibodies [Table 3], but in the presence of “extra-criteria” manifestations [Table 4]) or a “Laboratory non-criteria APS”, given the presence of clinical criteria with persistently negative or low titer criteria aPL, but positive “extra-criteria” aPL [18].

Considering the above, the clinical team assumed a Catastrophic SNAPS diagnosis and the patient was treated accordingly, with good results. The patient will be kept under warfarin treatment indefinitely and will perform follow-up laboratorial tests, to look for positive aPL or other laboratorial changes that that confirms CAPS or evidence another diagnosis.

## Conclusion

CAPS is a rare form of APS that develops quickly, with multiorgan involvement, high mortality rate, and its approach often poses a multidisciplinary challenge.

This patient presented with fetal loss and multiple arterial and venous thrombosis, clinical manifestations highly suggestive of CAPS, that hardly fits into another diagnosis, but with persistently negative aPL.

Some APS patients may present with negative aPL due to either consumption or presence of other anti-phospholipid antibodies, not routinely tested and are referred to as SNAPS or “Laboratory non-criteria APS”.

We believe that this is a rare case of catastrophic SNAPS, that presented a favorable evolution under therapy with anticoagulation, plasmapheresis and rituximab.

## Data Availability

Not applicable.
